# Taurine attenuates lipid accumulation via the eCB-CB1 axis: evidence from adipose metabolomics in HFD-fed mice and 3D adipocyte spheroids

**DOI:** 10.3389/fnut.2026.1782392

**Published:** 2026-03-06

**Authors:** Qingjie Wu, Yuxin Shao, Lin Zhu, Xinzhe Guo, Shengquan Mi, Yanzhen Zhang, Ping Chang, Changying Xie, Junxia Guo

**Affiliations:** 1 College of Biochemical Engineering, Beijing Union University, Beijing, China; 2 Beijing Key Laboratory of Bioactive Substances and Functional Food, Beijing Union University, Beijing, China

**Keywords:** cannabinoid receptor type 1, endocannabinoid, lipid accumulation, obesity, taurine

## Abstract

**Introduction:**

Obesity, driven by adipose tissue dysfunction, is a major global health challenge and a key contributor to metabolic disorders. Although taurine shows anti-obesity potential, its precise mechanisms for attenuating adipocyte lipid accumulation remain unclear.

**Methods:**

In this study, high-fat diet (HFD)-induced obese mice were treated orally with taurine (700 mg/kg/day) for 14 weeks. Systemic obesity-related parameters were evaluated, with a focus on epididymal white adipose tissue (eWAT). UPLC-MS-based metabolomics combined with multivariate analysis was employed to characterize metabolic alterations in eWAT. Additionally, 3T3-L1 adipocyte spheroids were treated with taurine (0–0.5 mM), either alone or in combination with the cannabinoid receptor type 1 (CB1) agonist CP55940 or antagonist AM6545, to assess its effects on lipid accumulation and underlying mechanisms.

**Results:**

Focusing on adipose tissue, taurine treatment effectively countered HFD-induced metabolic disturbances, particularly by suppressing epididymal fat mass accumulation and ameliorating adipocyte hypertrophy. Metabolomic profiling of eWAT revealed that taurine treatment reversed 15 out of 35 metabolic alterations, including the reduction of three anandamide (AEA) precursors, implying that taurine may alter endocannabinoid (eCB) biosynthesis by limiting precursor availability. Moreover, taurine suppressed lipid accumulation by inhibiting CB1 signaling, a mechanism supported by downregulation of lipogenic genes (including *Srebf1*, *Acaca*, *Cd36*, and *Pparg*) and upregulation of lipolytic genes (including *Pnpla2*, *Lipe*, and *Ppargc1a*).

**Conclusion:**

Collectively, our findings demonstrate that taurine exerts its anti-obesity effects partially via modulation of eCB-CB1 signaling, coordinately inhibiting lipogenesis and promoting lipolysis, thereby highlighting its therapeutic potential for obesity management.

## Graphical abstract

**Figure:**
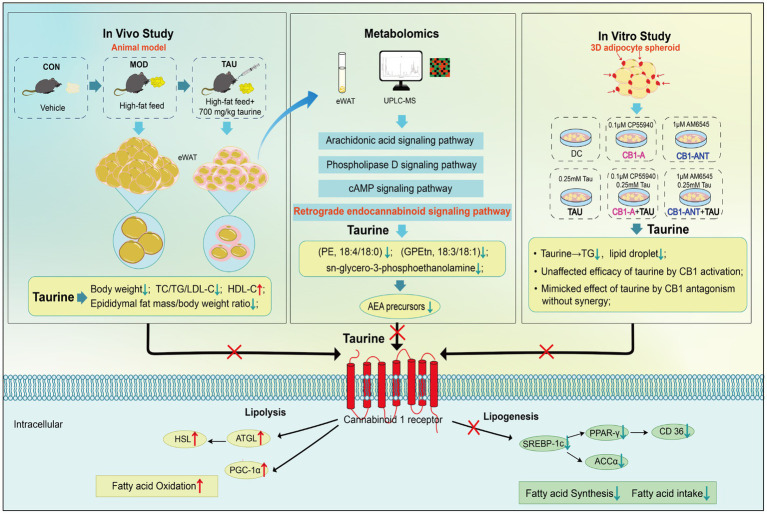

## 1 Introduction

Obesity is a global health challenge that increases the risk of cardiovascular disease, type 2 diabetes, and non-alcoholic fatty liver disease (1–3). According to a 2025 Lancet analysis spanning 204 countries and territories, the prevalence of overweight and obesity among adults rose steadily from 1990 to 2021, already affecting 1.00 billion men and 1.11 billion women in 2021. If current trends continue, this number is projected to reach 3.8 billion adults—more than half of the global adult population—by 2050 (4). Urgent action is needed to slow this trend and reduce the burden of type 2 diabetes and related chronic conditions. However, current pharmacotherapies often have significant adverse effects (5, 6), which promotes the search for safer alternatives, particularly from natural sources.

Taurine (2-aminoethanesulfonic acid; Tau) is a conditionally essential amino acid that is abundant in mammalian plasma and metabolically active tissues, such as the liver, heart, and white adipose tissue (WAT) (7, 8). Although synthesized endogenously, the majority of taurine is obtained exogenously through diet, particularly from seafood (9). Clinical evidence indicates that circulating taurine levels are significantly lower in individuals with obesity and/or diabetes compared to lean healthy controls (10–12). Taurine supplementation not only elevates plasma taurine and adiponectin levels, but also reduces body weight and ameliorates dyslipidemia—specifically lowering triglycerides (TG), total cholesterol (TC), and atherosclerotic index (13). Both rodent (14, 15) and human (16, 17) studies report that taurine administration significantly reduces adiposity without serious adverse effects, even at high doses. Thus, taurine is a safe, well-tolerated candidate for combating obesity.

Mechanistically, taurine regulates adipose lipid homeostasis by suppressing lipogenesis and promoting fatty acid oxidation. It suppresses *de novo* lipogenesis by downregulating the lipogenic transcription factors sterol regulatory element-binding protein-1c (SREBP-1c) and peroxisome proliferator-activated receptor-γ (PPAR-γ), and the enzyme fatty acid synthase (FAS) (18), while activating catabolic programs via upregulation of peroxisome proliferator-activated receptor-α (PPAR-α), peroxisome proliferator-activated receptor-γ coactivator-1α (PGC-1α), and 5’-AMP-activated protein kinase (AMPK), thereby accelerating fatty acid oxidation and lipid turnover (19, 20). Additionally, taurine promotes mitochondrial biogenesis and thermogenic capacity, as evidenced by the beiging of inguinal WAT and upregulation of PGC-1α and uncoupling protein 1 (UCP1) (21–25). Collectively, these findings cast taurine as a pivotal regulator of energy balance, lipid flux, and obesity-related metabolic dysfunction.

Notably, the adipose endocannabinoid (eCB) system—centered on cannabinoid receptor type 1 (CB1) and activated by its principal ligands, 2-arachidonoylglycerol (2-AG) and anandamide (AEA)—serves as a critical regulator of these lipid metabolic programs (26). CB1 activation stimulates lipoprotein lipase activity and fatty acid uptake, drives *de novo* lipogenesis via upregulation of SREBP-1c and PPAR-γ, suppresses AMPK-mediated fatty acid oxidation, and facilitates adipocyte differentiation, thereby expanding WAT and fostering insulin resistance (27–29). Conversely, peripheral CB1 antagonism normalizes metabolic defects in rodents by restoring leptin sensitivity, alleviating hyperleptinaemia, and improving glucose and lipid homeostasis, alongside resolving hepatic steatosis and fibrosis (30–33). This striking convergence on identical molecular targets (SREBP-1c, FAS, PPAR-γ, AMPK, and PGC-1α) suggests the eCB-CB1 axis to be a plausible upstream mediator of taurine’s metabolic benefits.

In obesity, adipose tissue exhibits sustained dysregulation of eCBs homostasis, characterized by elevated 2-AG and AEA (34, 35), along with upregulation of the biosynthetic enzymes diacylglycerol Lipase-α/β (DAGL-α/β) and N-acyl phosphatidylethanolamine phospholipase D (NAPE-PLD) (36). This pathological eCB overactivity drives CB1 hyperactivation, establishing a feed-forward loop that exacerbates adipocyte hypertrophy and ectopic lipid accumulation. Circulating eCBs levels correlate positively with every hallmark of metabolic dysfunction—visceral adiposity, insulin resistance, dyslipidaemia, and hepatic steatosis (34, 37–40). Importantly, Guo et al. (41) demonstrated that taurine supplementation reduced elevated hepatic Diacylglycerols (DGs) in high-fat diet (HFD)-fed mice. Given that DGs are direct precursors of 2-AG, the principal eCB activating CB1, these data position taurine as a potential modulator of adipose eCB signaling. However, whether taurine exerts its metabolic benefits through regulating the eCB-CB1 axis remains to be elucidated.

Here, we integrated untargeted UPLC-MS metabolomics of epididymal WAT (eWAT) from HFD-fed mice with mechanisms studies in three-dimensional (3D) adipocyte spheroids to determine whether taurine restores lipid metabolism by antagonizing or functionally desensitizing adipocyte cannabinoid receptors. The findings are expected to identify novel molecular targets and provide a theoretical framework for developing taurine-enriched functional foods or next-generation anti-obesity therapeutics.

## 2 Materials and methods

### 2.1 Chemicals and assay kits

Taurine (107-35-7) was from Shanghai Aladdin Biochemical Technology. The main chemicals and assay kits used in animal experiments are the same as those in Guo et al. (41). Methanol (67-56-1), acetonitrile (75–05-8), and Water (7732-18-5) were from Fisher Scientific (Pittsburgh, PA, United States). Formic Acid was from CNW Technologies GmbH. The TG (A110-1-1) kit was from Nanjing Jiancheng Bioengineering Institute (Jiangsu, China). Dexamethasone (HY-14648), insulin (HY-P0035), rosiglitazone (HY-17386), 3-Isobutyl-1-methylxanthine (IBMX; HY-12318), thyroxine (T3; HY-A0070AR), and AM6545 (CB1 antagonist, HY-110206) were from MedChemExpress LLC (China). CP55940 (CB1 agonist, C1112) was from Sigma-Aldrich (Shanghai) Trading Co., Ltd.

### 2.2 Animal experiment

The animal experiment was performed exactly as described in Guo et al. (41). All animals were housed under controlled conditions (22 ± 2 °C, 50 ± 10% relative humidity) on a 12-h light/dark cycle, with free access to food and water. Each cage housed two to three animals to promote social interaction while ensuring equal ad libitum feeding. For obesity induction, male C57BL/6 J mice (8 weeks old, 21 ± 1 g) were fed an HFD (41% kcal from fat, 43% kcal from carbohydrate, 16% kcal from protein) for 14 weeks. Mice were randomly assigned to three groups (n = 9 per group): (1) control group (CON), receiving normal diet (10% kcal from fat, 70% kcal from carbohydrate, 20% kcal from protein) and vehicle (distilled water); (2) HFD group (MOD), receiving HFD and vehicle; (3) TAU group, receiving HFD and taurine (TAU, 700 mg/kg). Tauirne or vehicle was administered daily by oral gavage beginning on the first day of HFD feeding.

Randomization was performed using a random number table to minimize bias. Throughout the intervention period, food intake and body weight were monitored daily. After 14 weeks, mice were fasted for 10 h, anesthetized with 4% isoflurane, and immediately euthanized by cervical dislocation. Serum and epididymal fat mass were collected for subsequent biochemical, histological, and metabolomic analyses. Epididymal fat masses were rinsed in ice-cold saline, blotted dry, and weighed to determine their wet weights. The epididymal fat mass-to-body weight ratio was calculated as (epididymal fat weight/body weight) × 100%. Body weight, serum lipid, and adipose tissue H&E images are presented as phenotypic validation. All outcome assessment and data analysis were performed by investigators blinded to the group assignments. This study used only animal procedures approved by the Experimental Animal Ethics Committee of the Functional Test Center for Health Food, College of Arts and Sciences, Beijing Union University (Approval No. 20220301) (41).

### 2.3 Metabolomics sample preparation

Adipose tissue (50 ± 5 mg) was homogenized in 400 μL ice-cold methanol/water (4:1, v/v), sonicated for 30 min at 5 °C (40 KHz), and centrifuged at 12,000 rpm for 15 min. After activation of CNWBONDHD-C18 SPE cartridges, the eluates were collected, centrifuged at 15,000 rpm for 20 min, and the supernatant was subjected to UPLC-MS analysis.

As part of system conditioning and quality control (QC), a pooled QC sample was prepared by mixing equal volumes of eWAT extracts from all individuals in each group (CON, MOD, and TAU). The QC sample was processed and analyzed identically to the study samples, with one injection every six analytical runs.

### 2.4 UPLC-MS conditions

Metabolites were separated on an ACQUITY UPLC HSST3 column (100 mm × 2.1 mm i.d., 1.8 μm; Waters, Milford, United States) at 40 °C, using a UHPLC-Orbitrap Exploris 240 system (Thermo Fisher Scientific, Waltham, MA, United States). The autosampler tray was held at 4 °C, and 3 μL of each extract was injected. Gradient elution was performed using (A) water/acetonitrile (95:5, v/v) and (B) acetonitrile/isopropanol/water (47.5:47.5:5, v/v/v), both containing 0.1% formic acid. One pooled QC sample was analyzed after every six study samples to monitor system stability.

Detection was carried out on a Q Exactive Plus mass spectrometer (Thermo Fisher Scientific, Waltham, MA, United States) with electrospray ionization in positive (ESI^+^) and negative (ESI^−^) ion modes (70–1,050 m/z). Source conditions: spray voltage 3.4 kV (ESI^+^) or 3.0 kV (ESI^−^); heater temperature 350 °C; capillary temperature 320 °C; sheath gas 60 arbitrary units; auxiliary gas 20 arbitrary units; S-lens RF 70. Data were acquired and processed with Xcalibur 4.1 (Thermo Fisher Scientific). MS/MS data were acquired in data-dependent acquisition (DDA) mode.

### 2.5 Multivariate statistical analysis and identification of potential differential metabolites

This study employed untargeted metabolomics for relative quantitative comparison of metabolites across groups, rather than absolute concentrations. The UPLC-MS raw data were imported into Progenesis QI software (Waters Corporation, Milford, United States) for peak picking, noise filtering, and automatic alignment. Metabolic features were characterized by retention time (RT), mass-to-charge ratio (m/z), and sum-normalized peak area. Features with missing values in more than 20% of samples within any group were excluded, and remaining missing values were imputed using the minimum value method. The data were log10-transformed before multivariate statistical analysis.

Metabolite features were identified by matching the accurate mass, MS/MS fragment spectra, and isotope patterns against reliable databases such as HMDB and METLIN. Analytical quality was assessed using pooled QC samples; features with a relative standard deviation (RSD) of more than 30% across QCs were excluded. Principal component analysis (PCA) of QC samples showed tight clustering, indicating negligible instrumental drift. Consequently, no batch correction was applied. The processed data matrix was subjected to PCA and orthogonal partial least squares discriminant analysis (OPLS-DA). PCA revealed a distinct metabolic profile among the CON, MOD, and TAU groups. The OPLS-DA model was validated using 200 permutation tests. Differential metabolites were selected based on variable importance in projection (VIP) > 1.0 (from OPLS-DA) and *p* < 0.05 (Student’s *t*-test). Given the exploratory nature of this untargeted profiling, features were prioritized by VIP and nominal *p*-values without stringent false discovery rate (FDR) correction, consistent with established practice for hypothesis-generating metabolomics studies (42, 43).

### 2.6 Pathway analysis

Differential metabolites were subjected to enrichment analysis using the Python package Scipy stats against the KEGG database and subsequently analyzed in MetaboAnalyst 5.0 to identify obesity-related pathways modulated by taurine.

### 2.7 Culture of 3T3-L1 preadipocytes

3T3-L1 preadipocytes (CL-0006, Wuhan Pricella) were cultured in high glucose (4.5 g/L, Genview) containing 10% newborn calf serum (NCS, 24060201, Sijiqing) and 1% w/v antibiotics (100 U/mL penicillin-0.1 mg/mL streptomycin) at 37 °C with 5% CO_2_. Cells were passaged at approximately 70% confluence, and those between passages 4–8 with more than 90% viability were selected.

### 2.8 Spheroid fabrication

At 70% confluence, cells were trypsinized, counted, and resuspended in DMEM containing 10% NCS and 0.25% methylcellulose (9004-67-5, Solarbio) to a density of 8,000 cells per 15 μL. Fifteen-microliter drops were placed on the inverted lid of a 10-cm Petri dish containing 6 mL sterile PBS to prevent evaporation. After reattaching the lid, hanging drops were formed and incubated at 37 °C with 5% CO_2_ for 48 h, allowing cells to aggregate into compact spheroids for subsequent experiments.

### 2.9 Adipogenic differentiation of spheroids

**Figure 1:**
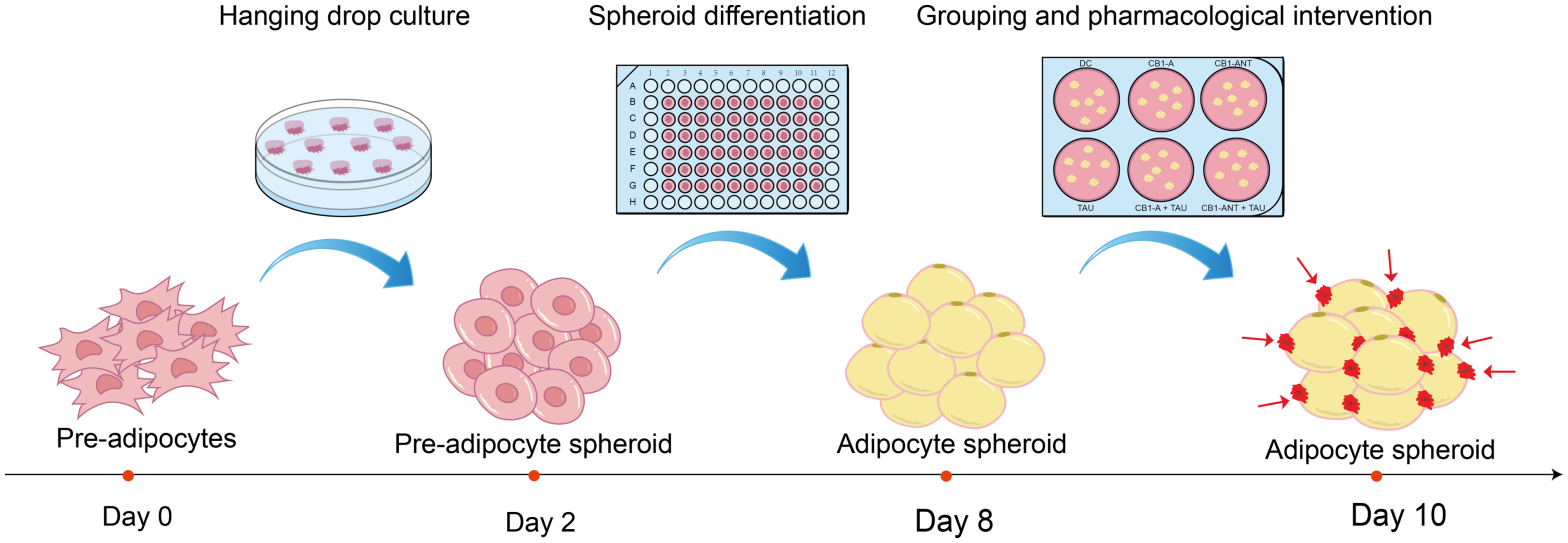
Schematic overview of 3T3-L1 adipocyte spheroids generation and drug treatment. The process involves spheroids formation by the hanging drops culture, differentiation into adipocyte spheroids, followed by grouping and pharmacological intervention.

Spheroids were maintained in 96-well plates pre-coated with 60 μL of 1.5% (w/v) agarose to preserve their 3D architecture. Adipogenic differentiation was induced for 4 days in DMEM supplemented with 10% Fetal Bovine Serum (FBS, 10099141C, Invitrogen), 1% antibiotics, 1 μM dexamethasone, 1 μg/mL insulin, 0.5 mM IBMX, 10 μM rosiglitazone, and 10 nM T3 (200 μL per well, refreshed every 48 h), followed by 2 days in maintenance medium (DMEM/10% FBS, 1% antibiotics, 1 μg/mL insulin). On day 6, spheroids were randomized into experimental groups and cultured for an additional 2 days: differentiated control (DC) receiving maintenance medium only; taurine at 0.1, 0.25, or 0.5 mM (TAU-L, TAU-M, TAU-H); CB1 agonist (CB1-A, CP55940, 0.1 μM); CB1 antagonist (CB1-ANT, AM6545, 1 μM); and the corresponding combinations with 0.25 mM taurine (CB1-A + TAU, CB1-ANT + TAU). Undifferentiated controls (UDC) were maintained in DMEM containing 10% NCS for 8 days with medium exchange every 48 h. A schematic overview of the experimental design is presented in Figure 1. The effective concentrations of agonists/antagonists (determined by cAMP response plateaus) were selected following CCK-8 cytotoxicity screening (Supplementary Figure S1).

### 2.10 Lipid morphology

Lipid droplets and nuclei were visualized by sequential staining with BODIPY 493/503 (HY-W090090, MCE) and DAPI (GD3410, Genview). Spheroids were fixed in 4% (w/v) paraformaldehyde for 1 h, washed three times with PBS, and incubated with the dyes according to the manufacturer’s protocols, with PBS rinses between steps. Images were acquired on a Leica fluorescence microscope.

### 2.11 TG level determination

Intracellular TG was quantified using a commercial kit, normalized to total protein (BCA assay, BCA02, Beijing Dingguo Biotechnology), and expressed as μmol per gram protein.

### 2.12 Gene expression

Total RNA was isolated from the mouse eWAT and 3T3-L1 adipocyte spheroids with TRIzol reagent (10,057,841, Invitrogen). eWAT samples were dissected, snap-frozen, and homogenized before extraction. For spheroids, about 150 adipocyte spheroids were pooled and lysed per sample. Concentration and purity (A260/A280 ≈ 2.0, A260/A230 > 1.8) were verified using a NanoDrop spectrophotometer (Thermo Scientific). cDNA was synthesized from 1 μg of RNA with NovoScript Plus All-in-one SuperMix (E047, Novoprotein). qPCR was performed in triplicate on a Q9604 system (BioGenerator) with GS AntiQ SYBR Green Fast Mix (SQ410, Genesand). Relative expression was calculated by the 2^(-ΔΔCt) method, normalized to 18S rRNA. Primers sequences are listed in Table 1.

**Table 1:** The primers sequences.

Target gene	Sequence (5′ → 3′)
*Rn18s*	F: AGAGGGAAATCGTGCGTGAC R: CAATAGTGATGACCTGGCCGT
*Cnr1*	F: TGCACAAGCACGCCAATAAC R: ACAGTGCTCTTGATGCAGCTTTC
*Srebf1*	F: ACACAGCAACCAGAAACTCAAG R: AGTGTGTCCTCCACCTCAGTCT
*Acaca*	F: GGCAATGACATCACATACCG R: CAGTCCGATTCTTGCTCCAC
*Cd36*	F: ATGGGCTGTGATCGGAACTG R: GTCTTCCCAATAAGCATGTCTCC
*Pparg*	F: GACCACTCGCATTCCTTTGACA R: ATCGCACTTTGGTATTCTTGGA
*Lipe*	F: GCTGGGCTGTCAAGCACTGT R: GTAACTGGGTAGGCTGCCAT
*Pnpla2*	F: AGGACAGCTCCACCAACATC R: TGGTTCAGTAGGCCATTCCT
*Ppargc1a*	F: AACCTTAAGTGTGGAACTCTCTGGA R: CGTTTAGTCTTCCTTTCCTCGTGT
*Faah*	F: GTGGATAGCCTGGCATTGTG R: GGAGTGGGCATGGTGTAGTTG

### 2.13 Statistical analysis

Data are expressed as mean ± SD. Intergroup differences were analyzed using unpaired two-tailed Student’s t-tests, and one-way ANOVA followed by Dunnett’s *post hoc* test, with *p* < 0.05 considered significant.

## 3 Results

### 3.1 Taurine ameliorates obesity-related metabolic dysregulation and adipose tissue expansion

**Figure 2:**
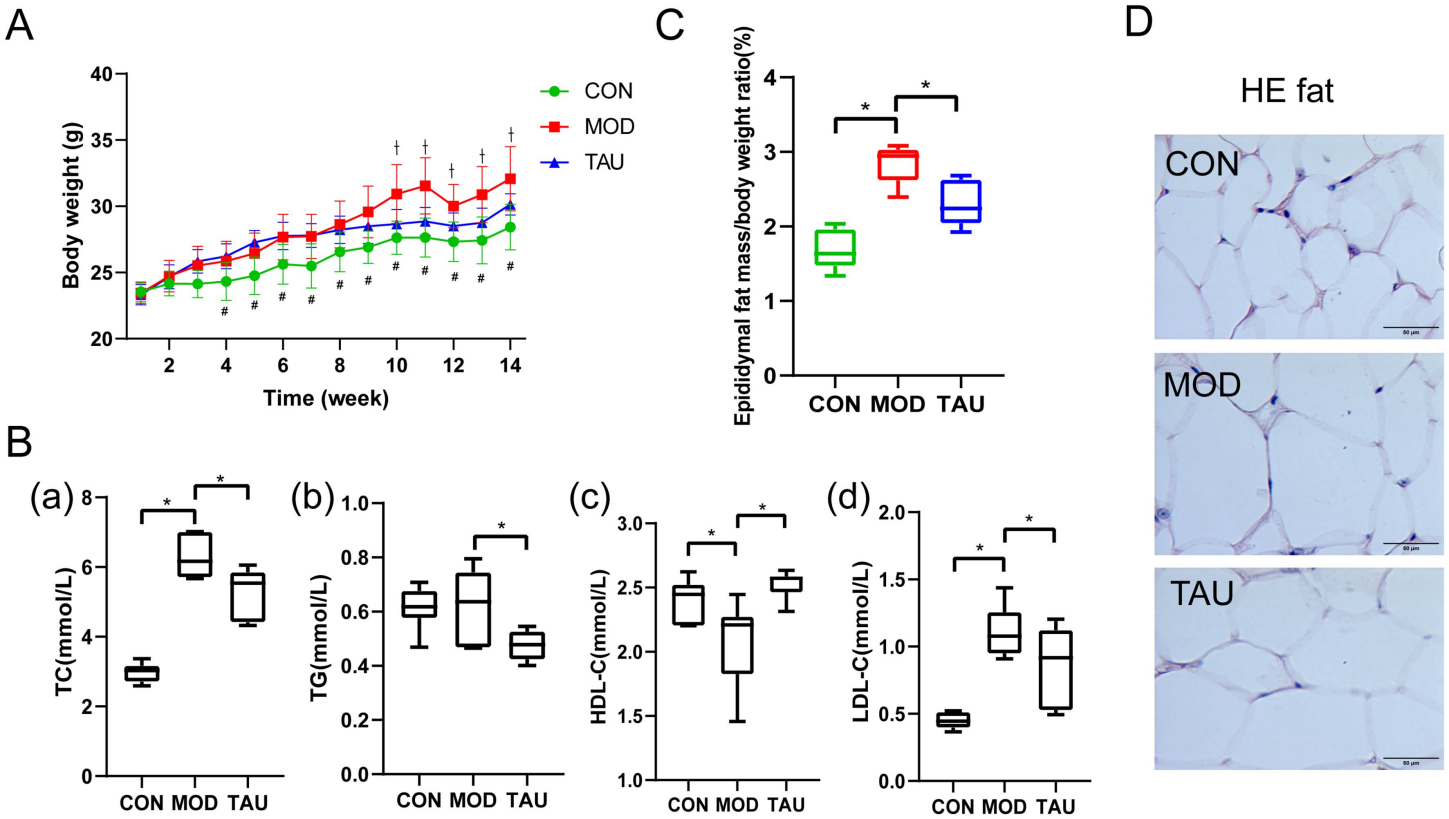
Taurine ameliorates obesity-associated metabolic phenotypes. **(A)** Body weight; **(B)** serum lipid profiles; **(C)** epididymal fat mass/body weight ratio; **(D)** representative H&E staining images of epididymal white adipose tissue (eWAT). Data in panels **(A,B,D)** were previously published in Guo et al. (41). In **(A)**, ^#^*p* < 0.05 the MOD vs. CON group; ^†^*p* < 0.05 the TAU vs. MOD group. In **(B,C)**, ^*^p < 0.05 between indicated groups.

Taurine administration had no significant effect on food intake throughout the treatment period, consistent with our previous report (Supplementary Figure S2) (41). Chronic taurine supplementation in obese mice significantly attenuated HFD-induced body weight gain (Figure 2A) and ameliorated the dyslipidemic profile (Figure 2B), marked by reductions in serum TC, TG, and low-density lipoprotein cholesterol (LDL-C) and an increase in high-density lipoprotein cholesterol (HDL-C). The improvement in adipose tissue morphology, as shown by the attenuation of adipocyte hypertrophy in H&E staining (41), was also confirmed (Figure 2D).

To further quantify the anti-obesity effect of taurine directly at the adipose tissue level, we measured the epididymal fat mass-to-body weight ratio. This analysis revealed that taurine treatment significantly suppressed the HFD-induced increase in epididymal fat mass (Figure 2C). The reduction in this fundamental adiposity metric further supports the improved tissue morphology (Figure 2D), collectively indicating that taurine ameliorates adipose tissue expansion. These findings establish adipose tissue as a target of taurine. Accordingly, we employed adipose tissue metabolomics and the 3D adipocyte models to elucidate the underlying mechanisms.

### 3.2 Taurine lowers endocannabinoid precursors in adipose tissue of obese mice

#### 3.2.1 Comprehensive quality control and metabolic feature profiling

Representative total ion chromatograms (TIC) of eWAT samples in both ESI^+^ and ESI^−^ modes, acquired by UPLC-MS, are presented in Supplementary Figure S3 to visualize metabolic profiles and assess the effect of taurine in obese mice. Additionally, the overlaid TICs of QC samples, prepared from pooled eWAT extracts across all groups, are shown in Supplementary Figure S4, confirming the analytical stability and repeatability throughout the run. All subsequent data indicate directional trends in metabolite abundance.

To further evaluate data quality, the RSD of all detectable peaks in the QC samples was calculated. As summarized in Supplementary Figure S5, more than 96.7% (Pre QC) and 92.5% (Raw QC) of metabolic features exhibited RSD values below 30%, demonstrating high reproducibility and reliability of the dataset and supporting its suitability for subsequent statistical analysis.

Following preprocessing—including missing value screening, normalization, QC-based validation, and data transformation—a total of 1733 metabolic features were extracted from all samples. These comprised 824 in ESI^+^ and 909 in ESI^−^ mode, as detailed in Table 2.

**Table 2:** Total ion number and database identification.

Ion mode	All peaks	Identified metabolites	Metabolites in library	Metabolites in KEGG
Pos	6,564	824	781	436
Neg	8,370	909	870	530

#### 3.2.2 Multivariate statistical analysis reveals altered metabolic profiles

**Figure 3:**
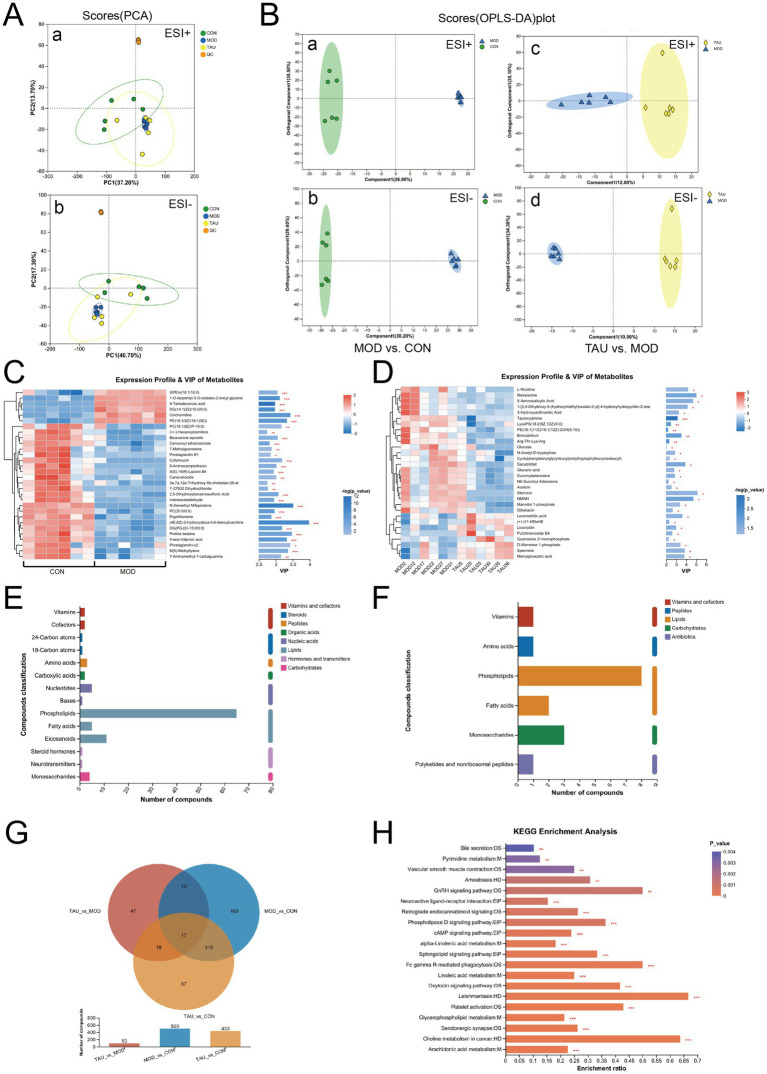
Comprehensive metabolomics analysis of eWAT. **(A)** Principal component analysis (PCA) score plots in ESI^+^ (a) and ESI^−^ (b). **(B)** Orthogonal partial least squares discriminant analysis (OPLS-DA) score plots for pairwise comparisons in both ESI^+^ and ESI^−^ ionization modes: MOD vs. CON (a, b) and TAU vs. MOD (c, d). **(C,D)** Hierarchical clustering heatmaps of differential metabolites for **(C)** MOD vs. CON and **(D)** TAU vs. MOD. **(E,F)** Classification of differential compounds for **(E)** MOD vs. CON and **(F)** TAU vs. MOD. **(G)** Venn diagram illustrating the overlap of differential metabolites among the three groups. **(H)** KEGG pathway enrichment analysis for differential metabolites. *p < 0.05, **p < 0.01, ***p < 0.001. Metabolite prioritization was based on a combination of a variable importance in projection (VIP) score > 1.0 and a *p*-value < 0.05.

Unsupervised PCA was initially performed to visualize the overall metabolic distribution among the CON, MOD, and TAU groups. While the QC samples remained tightly clustered, indicating sustained analytical stability (Figure 3A), distinct group-specific metabolic signatures were observed alongside moderate separation between groups. This indicates that global metabolic differences among groups are modest at the unsupervised level, likely reflecting inherent biological variability. Subsequently, pairwise OPLS-DA models were constructed to compare metabolic profiles between groups. The MOD vs. CON comparison yielded a robust model (*R*^2^*Y* = 0.967, *Q*^2^ = 0.706), indicating substantial metabolic perturbations induced by HFD. Similarly, the TAU vs. MOD comparison demonstrated strong predictive ability (*R*^2^*Y* = 0.896, *Q*^2^ = 0.770), reflecting taurine-mediated metabolic alterations. Permutation tests (*n* = 200) confirmed model validity, as the regression lines of both *Q*^2^ and *R*^2^*Y* intersected the *Y*-axis below zero (*p* < 0.05), effectively ruling out overfitting. Score plots revealed clear separation in both comparisons (Figure 3B).

#### 3.2.3 Screening and characterization of differential metabolites

Based on the OPLS-DA model, metabolites with VIP > 1.0 and a *t*-test *p*-value<0.05 were selected as significant contributors to inter-group differences. The clustering heatmaps of these differential metabolites clearly distinguished the MOD vs. CON groups (Figure 3C) and TAU vs. MOD groups (Figure 3D), highlighting the top 30 VIP-ranked metabolites in each comparison. These visualizations revealed distinct, group-specific metabolite signatures.

#### 3.2.4 Classification of differential metabolites

A total of 500 significantly altered metabolites were identified between groups MOD and CON, primarily belonging to glycerophospholipids (GPs), eicosanoids, fatty acids, and monosaccharides (Figures 3E,G). In contrast, 93 metabolites were significantly altered between groups TAU and MOD (Figures 3F,G), mainly including GPs, monosaccharides, fatty acids, and amino acids.

#### 3.2.5 Identification of common altered metabolites

A total of 35 differential metabolites were listed across all comparisons and are detailed in Table 3, along with their corresponding RT, m/z, VIP values, and change trends. Notably, of the 17 metabolites consistently altered in both comparisons, HFD feeding increased 7 and decreased 10 in MOD versus CON mice; taurine supplementation reversed 15 of these changes (6/7 increased and 9/10 decreased metabolites) in eWAT.

**Table 3:** Significantly metabolites in adipose tissue samples.

No	Category	Metabolite	Mode	RT (min)	M/Z	VIP	Trend	
							MOD/CON	TAU/MOD
1	Glycerophospholipids	PE(18:4(6Z,9Z,12Z,15Z)/18:0)	Positive	7.28	762.50	1.56	Up	Down
2	Glycerophospholipids	GPEtn(18:3/18:1)	Positive	7.48	740.52	1.11	Up	Down
3	Sphingolipids	Sphinganine	Positive	6.17	302.31	1.83	Up	Down
4	Sphingolipids	SM(d18:1/14:0)	Negative	7.40	719.53	1.36	Up	Down
5	Fatty acids	Arachidonic acid	Negative	6.66	303.23	1.34	Down	Up
6	Fatty acids	Eicosapentaenoic Acid	Negative	6.56	301.22	1.62	Down	Up
7	Bile acids	24-NorUrsodeoxycholic Acid	Negative	6.76	399.25	1.89	Down	Up
8	Amino acids	L-2-Amino-3-oxobutanoic acid	Negative	1.03	116.04	1.17	Down	Up
9	Monosaccharides	Glucose	Positive	0.59	383.12	2.29	Up	Down
10	Other	Succinyladenosine	Positive	3.46	384.11	1.68	Up	Down
11	Other	Cromakalim	Negative	6.25	267.11	1.15	Down	Up
12	Other	Cilastatin	Negative	6.65	403.16	1.02	Down	Up
13	Other	Ibuprofen glucuronide	Negative	6.60	427.16	1.37	Down	Up
14	Other	Pulchinenoside B4	Positive	3.81	622.31	2.48	Down	Up
15	Other	(2-Acetyloxy-3-hydroxypropyl) (E)-octadec-9-enoate	Positive	6.60	421.29	1.00	Down	Up
16	Glycerophospholipids	LysoPC(20:0/0:0)	Negative	6.87	596.39	1.99	Down	Down
17	Amino acids	Glucaric acid	Positive	3.46	252.07	1.66	Down	Down
18	Diradylglycerols	DG(14:1(9Z)/15:0/0:0)	Negative	7.08	505.43	2.97	Up	–
19	Diradylglycerols	DG(22:6(4Z,7Z,10Z,13Z,16Z,19)/22:5(4Z,7Z,10Z,13Z,16Z)/0:0)	Positive	7.38	697.52	1.36	Up	–
20	Sphingolipids	Sphingosine 1-phosphate	Negative	6.42	424.25	2.19	Up	–
21	Glycerophospholipids	Sn-glycero-3-Phosphoethanolamine	Negative	0.59	214.0	1.66	–	Down
22	Fatty acids	Docosahexaenoic acid	Negative	6.60	327.23	1.82	Down	–
23	Fatty acids	Cervonoyl ethanolamide	Negative	6.43	371.26	2.81	Up	–
24	Fatty acids	Linoleic acid	Negative	6.69	279.23	1.11	Down	–
25	Eicosanoids	PG(i-15:0/LTE4)	Negative	7.18	872.47	1.31	Up	–
26	Eicosanoids	18-carboxy dinor Leukotriene B4	Negative	6.14	337.17	1.75	Up	–
27	Eicosanoids	DG(PGE2/0:0/8:0)	Positive	0.75	585.40	1.93	Up	–
28	Amino acids	N-hydroxy-L-tryptophan	Negative	4.41	659.25	2.48	Down	–
29	Amino acids	N-Acetyl-D-tryptophan	Negative	5.98	551.22	2.86	–	Down
30	Amino acids	Formiminoglutamic acid	Positive	0.66	157.06	2.04	Up	–
31	Amino acids	Tyrosylhydroxyproline	Positive	4.20	295.13	2.01	Down	–
32	Vitamin	24-Oxo-1alpha,25-dihydroxyvitamin D3	Negative	6.56	451.30	1.26	Down	–
33	Vitamin	Riboflavin (Vitamin B2)	Positive	4.49	337.15	1.85	Down	–
34	Vitamin	Pantothenic Acid(Vitamin B5)	Positive	3.40	220.12	1.63	–	Up
35	Organoheterocyclic compounds	Indoleacetaldehyde	Negative	5.80	204.07	2.94	Down	–

CON, the control group; MOD, the model group; TAU, the taurine group.

All metabolites were identified by matching accurate mass, MS/MS fragment spectra, and isotope patterns against HMDB and METLIN databases. Given the non-targeted nature of the metabolomics analysis and the lack of authentic chemical standards, all metabolite identifications represent putative annotations (MSI Level 2). Accordingly, biological interpretations were performed cautiously, primarily at the pathway level.

#### 3.2.6 Pathway enrichment analysis

The KEGG pathway enrichment analysis revealed that the 15 reversed differential metabolites were significantly enriched in several pathways, with the retrograde endocannabinoid signaling pathway being among the most prominently impacted (Figure 3H). Notably, three candidate metabolites among these top 15 common differential metabolites—Glycerophosphoethanolamine (GPEtn, 18:3/18:1), phosphatidylethanolamine (PE, 18:4/18:0), and arachidonic acid (AA)—participate in eCB biosynthesis (Table 3). Their MS/MS spectra have been provided in Supplementary Figure S6. All three metabolites exhibited HFD-driven perturbations (GPEtn and PE increased, AA decreased) that were reversed by taurine supplementation, suggesting them as key precursors whose taurine-mediated restoration implies suppression of eCB synthesis. Moreover, HFD elevated levels of key eCB pathway components, including the direct synthesis substrates DGs (DG (22:6/22:5/0:0) and DG (14:1/15:0/0:0)) and downstream sphingolipid mediators sphingosine-1-phosphate and sphinganine, which were not reversed by taurine. However, taurine did markedly reduce sn-glycero-3-phosphoethanolamine, a molecule convertible to PE.

In summary, our metabolomics analysis revealed that taurine supplementation reversed HFD-induced alterations in 15 specific metabolites, with a pronounced impact centered on the endocannabinoid signaling through modulation of precursor availability.

### 3.3 Taurine attenuates lipid accumulation in differentiating 3T3-L1 spheroids via CB1 regulation

**Figure 4:**
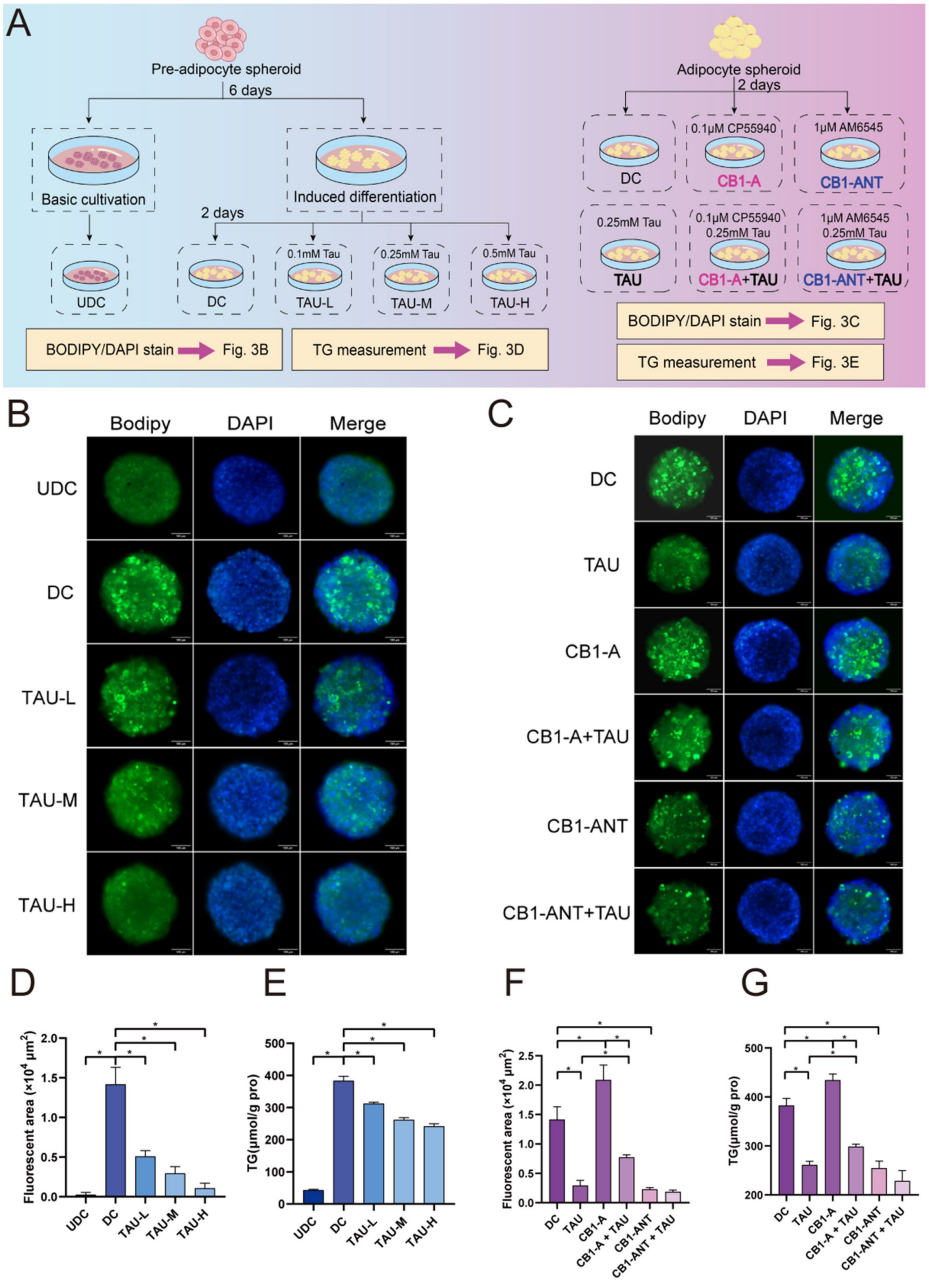
Taurine attenuates lipid accumulation in differentiated 3 T3-L1 spheroids via CB1 regulation. **(A)** Schematic of the experimental design. **(B)** Representative fluorescence images of 3 T3-L1 spheroids. From top to bottom: undifferentiated control spheroids (UDC), differentiated control spheroids (DC), and differentiated spheroids treated with taurine at concentrations of 0.1 mM (Tau-L), 0.25 mM (Tau-M), and 0.5 mM (Tau-H). Nuclei were stained with DAPI (blue), and lipid droplets were stained with BODIPY (green). **(C)** Representative fluorescence images of differentiated spheroids under various treatments: differentiated control (DC), taurine-treated (TAU), CP-55940 (CB1 agonist)-treated (CB1-A), CP-55940 and taurine co-treated (CB1-A + TAU), AM6545 (CB1 antagonist)-treated (CB1-ANT), and AM6545 and taurine co-treated (CB1-ANT + TAU). Staining is as in **(B)**. **(D,E)** Quantification of the fluorescence area and intracellular TG from **(B)**. **(F,G)** Quantification of the fluorescence area and intracellular TG from **(C)**. Data are presented as mean ± SD. *p < 0.05.

Based on the morphological and metabolomics alterations observed in HFD-fed mice, we employed 3T3-L1 adipocyte spheroids to further elucidate the mechanisms by which taurine regulates lipid metabolism. A schematic overview of the experimental design is presented in Figure 4A.

As shown in Figures 4B,D,E, differentiated control spheroids (DC) exhibited markedly enlarged lipid droplets (BODIPY-positive staining), increased fluorescence area, and elevated intracellular TG content compared to undifferentiated controls (UDC), confirming successful adipogenic maturation. Corroborating the *in vivo* findings in HFD-fed mice, taurine treatment dose-dependently attenuated these adipogenic markers in differentiated spheroids, with lipid droplet size, fluorescence area, and TG level all progressively decreasing at increasing concentrations (0.1, 0.25, and 0.5 mM, TAU-L~H; 48 h), demonstrating effective suppression of lipid accumulation.

To determine whether CB1 signaling contributes to this lipid-lowering effect of taurine, we performed pharmacological intervention experiments in differentiated adipocyte spheroids, assessing lipid accumulation by BODIPY staining (qualitative and quantitative fluorescence analysis) and intracellular TG content measurement. Taurine (0.25 mM, 48 h) significantly reduced lipid droplets (Figure 4C), fluorescence area (Figure 4F), and intracellular TG levels (Figure 4G), indicating potent attenuation of lipid accumulation. Activation of CB1 with CP55940 (0.1 μM, CB1-A) increased lipid accumulation compared to vehicle controls. Notably, the lipid-lowering efficacy of taurine was partially attenuated under CB1 activation, as evidenced by diminished reductions in both fluorescence area and TG content, suggesting that taurine acts, at least in part, through CB1-dependent mechanisms. In contrast, selective CB1 blockade with AM6545 (1 μM, CB1-ANT) reduced lipid accumulation to an extent comparable to taurine alone across all measured parameters. Co-treatment with taurine and CB1-ANT did not produce further reduction, indicating saturation of the lipid-lowering response. Collectively, these findings demonstrate that taurine suppresses adipocyte lipid accumulation through partially CB1-dependent mechanisms.

### 3.4 Taurine regulates mRNA expression of adipogenic and lipolysis-related factors by inhibiting CB1

**Figure 5:**
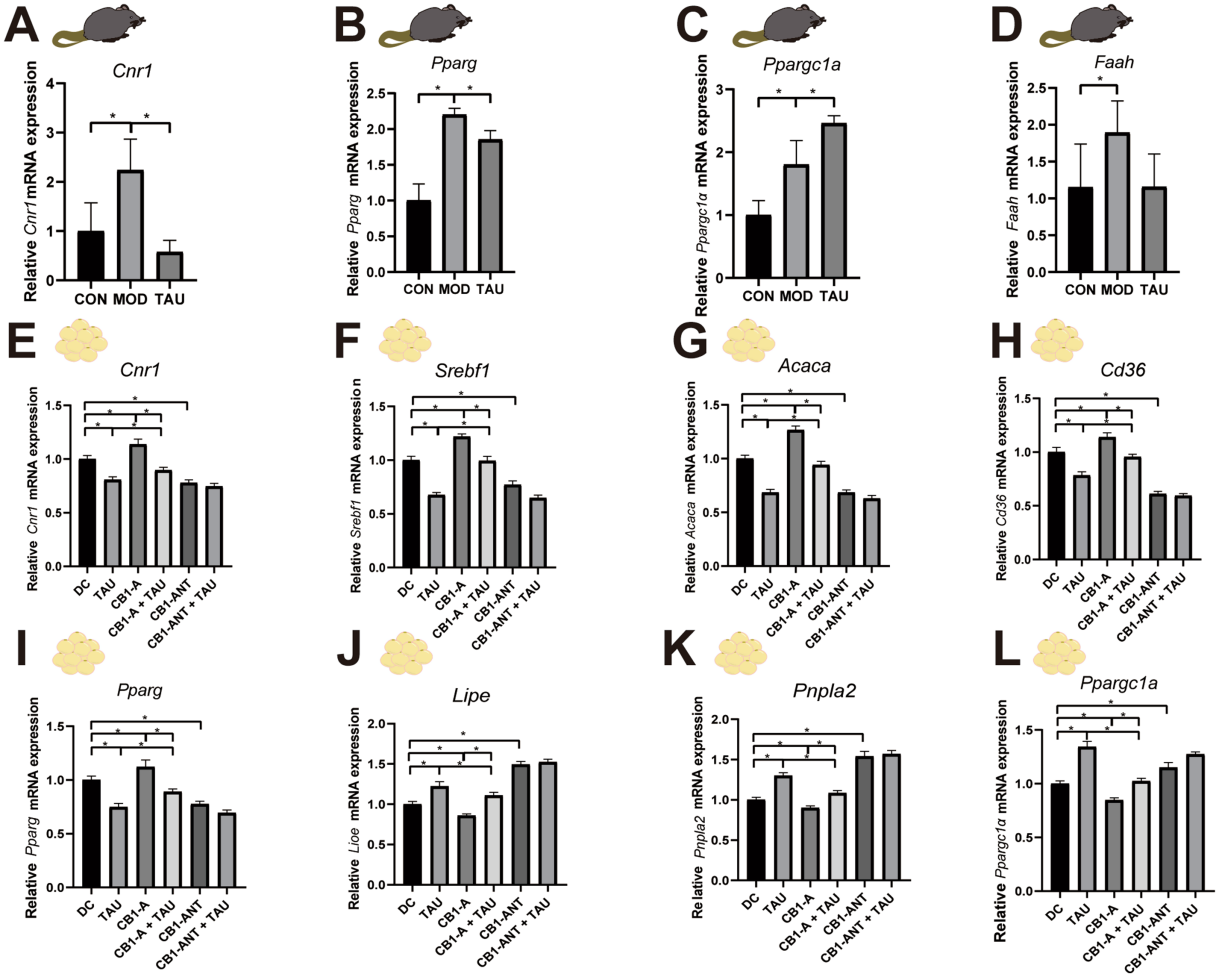
Taurine regulates adipogenic and lipolytic gene networks through CB1 inhibition. **(A–D)** mRNA expression levels of key regulatory genes in adipose tissue of HFD-induced mice: **(A)** *Cnr1*, **(B)** *Pparg*, **(C)** *Ppargc1a*, and **(D)** *Faah*. **(E–L)** mRNA expression profiles of adipogenesis and lipolysis-related genes in 3T3-L1 adipocyte spheroids: **(E)** *Cnr1*, **(F)** *Srebf1*, **(G)** *Acaca*, **(H)** *Cd36*, **(I)** *Pparg*, **(J)** *Lipe*, **(K)** *Pnpla2*, and **(L)** *Ppargc1a*. *p < 0.05.

Numerous studies have reported markedly increased CB1 levels in obese individuals (34, 35, 44). Consistently, mRNA expression of *Cnr1* and the eCB-degrading enzyme fatty acid amide hydrolase (*Faah)* was also observed to be significantly elevated in the MOD group relative to CON (Figures 5A,D). Given the established crosstalk between the eCB system and adipogenic metabolism, we concurrently quantified *Pparg* and *Ppargc1a* expression. Correspondingly, the levels of the lipogenic regulator *Pparg* and the master driver of fatty acid oxidation, *Ppargc1a*, were also upregulated in the MOD group (Figures 5B,C). Taurine supplementation substantially normalized *Cnr1* levels, partially reduced *Pparg* expression, and further enhanced *Ppargc1a* mRNA levels beyond those in the MOD group, while it had no significant effect on *Faah* expression. The coordinated effect of taurine on the CB1 pathway and these opposing metabolic phenotypes (*Pparg* downregulation and *Ppargc1a* upregulation) prompted us to determine the underlying cellular mechanisms, specifically whether CB1 mediates these effects, in a more controlled system.

In adipocytes, lipid accumulation represents a balance between anabolism (governed by adipogenic factors including *Srebf1*, *Acaca*, and *Pparg* (45) and fatty acid uptake via *Cd36*) and catabolism [via lipolytic enzymes *Lipe* and *Pnpla2*, and mitochondrial fatty acid oxidation regulated by *Ppargc1a* (46, 47)]. To delineate how taurine modulates this balance through the *Cnr1*, we assessed the expression of these key regulators in our 3D adipocyte model. As shown in Figure 5E, taurine treatment (TAU) significantly downregulated *Cnr1* mRNA expression, whereas the CB1 agonists (CB1-A) markedly increased it. Importantly, taurine counteracted the agonist-induced upregulation of *Cnr1*, demonstrating its inhibitory effect even under CB1-stimulated conditions. Moreover, the CB1 antagonist (CB1-ANT) alone similarly downregulated *Cnr1*, and no additive suppression was observed upon co-treatment with taurine, consistent with CB1-mediated action.

We next examined downstream transcriptional changes (Figures 5F–L). Taurine downregulated adipogenic genes (*Srebf1*, *Acaca*, *Pparg*) and the fatty acid transporter *Cd36*, while upregulating lipolytic genes (*Lipe*, *Pnpla2*) and *Ppargc1a*. Conversely, CB1 activation promoted adipogenic gene expression but suppressed lipolytic and oxidative transcripts. These effects were effectively counteracted by taurine. Furthermore, the CB1 antagonist alone largely recapitulated the regulatory pattern of taurine, and no synergistic effects were detected in combination treatments.

Together, these results demonstrate that taurine modulates adipogenic and lipolytic gene expression through a CB1-dependent pathway.

## 4 Discussion

Obesity, a global pandemic, contributes significantly to metabolic disorders primarily through dysfunctional adipose tissue. Taurine, a naturally occurring amino acid, exhibits anti-obesity potential and a favorable safety profile, yet the precise mechanisms by which it attenuates lipid accumulation in adipocytes remain elusive. This study identifies a novel role for taurine in combating obesity via CB1 signaling-dependent regulation of adipose tissue metabolism. Specifically, eWAT metabolomics analysis revealed that taurine treatment reversed 15 out of 35 molecular alterations, including the reduction of three AEA precursors, implying that taurine may suppress eCB biosynthesis by limiting precursor availability. Furthermore, using *in vitro* adipocyte spheroids, we found that taurine suppresses lipid accumulation by attenuating CB1 signaling, a mechanism corroborated by reduced lipogenic and enhanced lipolytic gene expression. Collectively, these results suggest that taurine is a potent therapeutic agent targeting the eCB-CB1 axis for the treatment of obesity.

In the present study, mice received taurine at a dose of 700 mg/kg via daily gavage for 14 weeks, which ensured more accurate dosing than administration via diet or drinking water. The mouse dose of 700 mg/kg corresponds to a human equivalent dose of approximately 57 mg/kg, which equates to a total dose of about 3.4 g for a 60 kg adult, based on body surface area conversion. It is important to note that the safety of taurine in humans has been established in clinical settings. Published studies report that even at relatively high oral doses, such as 3 g/day for 7 weeks or 6 g/day for 4 weeks, they showed a favorable safety profile (48). Consistent with these safety data, previous murine studies have employed taurine at doses of 500–1,000 mg/kg/day and reported beneficial metabolic effects without adverse outcomes (49). Collectively, these findings support the safety of our selected taurine dose for metabolic intervention.

A key finding from our animal studies is that taurine ameliorates lipid metabolic disorders by precisely modulating the eCB system in adipose tissue. Our metabolomics results revealed that an HFD elevated levels of key phospholipid metabolites, including PE, its derivative GPEtn, and DG, while concurrently depleting free AA. This profile is consistent with membrane phospholipid remodeling and increased eCB precursor availability for eCB synthesis (50, 51). The reduction in AA likely reflects its heightened conversion into inflammatory mediators (10, 52, 53); metabolomic data also showed elevated levels of AA-derived metabolites such as PG(i-15:0/LTE4), DG(PGE2/0:0/8:0), and 18-carboxy dinor Leukotriene B4, supporting increased AA utilization in eicosanoid pathways (54, 55). Therefore, AA decrease does not oppose eCB system upregulation but suggests augmented metabolic flux, aligning with reported eCB upregulation in obesity (56, 57). Crucially, taurine treatment selectively reversed this profile by normalizing PE/GPEtn and AA levels without altering DGs in eWAT, while our previous report suggests that taurine reversed DG abnormalities in the liver tissue of HFD mice (41), indicating that taurine has tissue or pathway specificity in its effects. This selective efficacy provides insight into the mechanism of taurine. The reversal by taurine of both PE and GPEtn—specific substrates for the NAPE-PLD-mediated biosynthesis for AEA (58, 59)—suggests a targeted effect on this branch of the eCB biosynthetic pathway. Furthermore, as a recognized anti-inflammatory agent, taurine may promote AA accumulation by lowering inflammatory factors and reducing the demand for AA in eicosanoids. In contrast, the lack of an effect on DGs, the primary precursor for 2-AG synthesis via DAGL (60), indicates that taurine does not broadly inhibit all eCB production but rather acts with specificity. This alteration in precursor lipid levels is further reinforced by the downregulation of CB1 mRNA following taurine intervention. Since elevated eCB signaling promotes adiposity and metabolic dysfunction (61, 62), our findings indicate that taurine alleviates obesity, at least in part, by attenuating the adipose tissue eCB-CB1 axis through limiting precursor availability and receptor expression.

In addition to the eCB pathway, we also found that taurine’s anti-obesity effects involve other processes, including the cAMP and phospholipase D signaling pathway, as well as GP and sphingolipid metabolism. These pathways do not operate in isolation but exhibit extensive crosstalk with the eCB signal network (63–65). For example, eCB inhibited the production of cAMP—a key second messenger for lipolysis—through the CB1 receptor (66). Furthermore, beyond serving as the source of eCB and its precursors (as previously mentioned), GPs and other membrane phospholipids contribute to the metabolic environment that supports the corrective effects of the eCB pathway in obesity. Therefore, taurine’s synergistic regulation of these pathways, including their extensive crosstalk with the eCB signaling, fosters a metabolic environment that inhibits lipogenesis and promotes lipolysis.

To elucidate the mechanistic actions of taurine in adipocytes, we employed a 3D spheroid culture system of 3 T3-L1 cells. This model recapitulates key aspects of the *in vivo* adipose tissue microenvironment, thereby enhancing adipocyte maturation and promoting lipid droplet formation and TG accumulation compared to 2D culture (50), thus providing a robust platform for our mechanistic studies. Treatment with taurine resulted in an expected and dose-dependent reduction of lipid droplets and TG levels, confirming taurine’s efficacy within this improved model. Furthermore, our results demonstrated that taurine suppresses lipid accumulation primarily through attenuating CB1 signaling, as evidenced by pharmacological studies utilizing both CB1 activation and blockade. Activation of the CB1 promotes lipogenesis and inhibits lipolysis (67–69). CB1 activation promotes fatty acid uptake, inhibits AMPK-mediated lipid oxidation, and promotes adipocyte differentiation. On the contrary, peripheral CB1 antagonism normalizes these defects, restores various indicators, and improves glucose and lipid status (27–29). Taurine is similar to CB1 antagonists in improving metabolic phenotype, which further demonstrates the regulatory effect of taurine on the activity of the eCB system. Thus, taurine ameliorates lipid accumulation in adipocytes by targeting the CB1, paving the way for its development as a novel anti-obesity therapeutic strategy.

**Figure 6:**
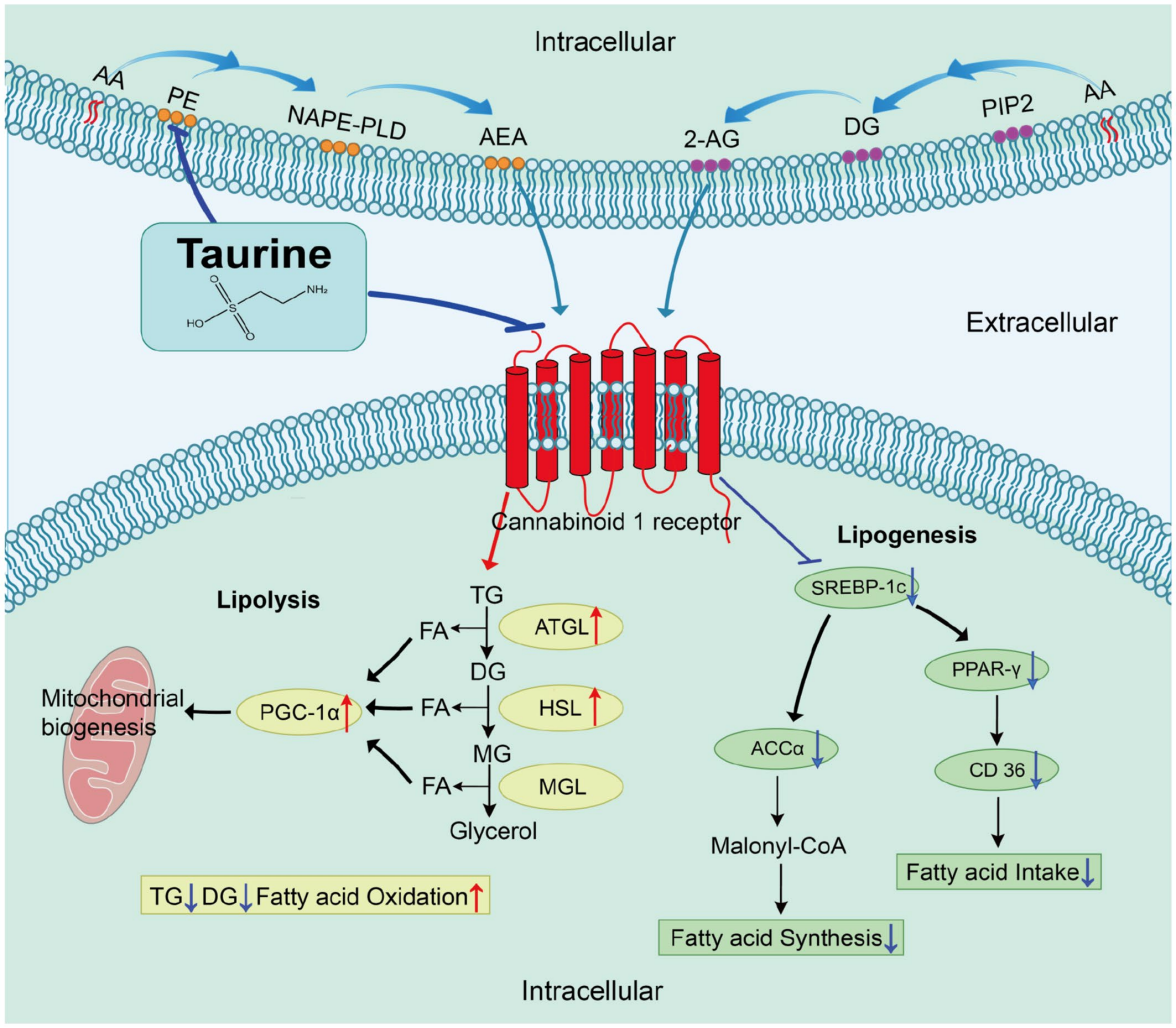
Working model: taurine regulates adipose lipid metabolism through partial modulation of the eCB-CB1 axis.

Elevated eCB levels and the subsequent CB1 activation are key hallmarks of obesity, driving lipid metabolic disorders through a well-characterized signaling axis (70). Specifically, activated CB1 promotes lipogenesis by upregulating *Srebf1* and *Acaca* (71, 72), enhances fatty acid uptake via *Cd36* (73), stimulates adipocyte differentiation through *Pparg* (74, 75), and suppresses lipolysis by inhibiting *Pnpla2* and *Lipe* (76, 77). Moreover, CB1 activation reduces mitochondrial fatty acid oxidation via downregulation of *Ppargc1a* (78). In this study, taurine’s reversal of the CB1 agonist-induced metabolic shift (from lipolysis to lipogenesis) provides functional evidence that taurine actively opposes CB1-mediated signaling. Furthermore, the finding that taurine does not synergistically enhance the lipid-catabolic response elicited by a CB1 inhibitor suggests convergence on shared downstream signaling events, consistent with taurine acting upstream of the receptor. This interpretation is supported by previous studies demonstrating that taurine downregulates lipogenic factors (*Srebf1, Acaca, Cd36*, and *Pparg* (79–82)) and upregulates critical lipolytic and oxidative mediators [such as *Pnpla2, Lipe,* and *Ppargc1a* (83, 84)]. Therefore, our results, as summarized in Figure 6, suggest that modulation of the eCB-CB1 axis represents a potential upstream mechanism through which taurine coordinates its broad transcriptional regulation of lipid storage and mobilization pathways.

While these findings implicate the eCB-CB1 axis in taurine’s metabolic effects, the precise molecular mechanisms warrant further investigation. Our conclusions are based on pharmacological interventions and precursor lipid alterations. However, direct assessment of CB1 receptor activation states (e.g., cAMP inhibition, receptor phosphorylation) and absolute quantification of eCB levels (AEA and 2-AG) by targeted LC–MS/MS were not performed and will be addressed in future studies. Consequently, whether taurine acts as a direct CB1 antagonist or indirectly modulates the pathway through upstream regulation remains to be elucidated. Future studies incorporating stable isotope-labeled internal standards for absolute quantification will address this gap.

## 5 Conclusion

Overall, our work reveals that taurine alleviates obesity by attenuating the adipose tissue eCB-CB1 axis. This inhibition coordinates a transcriptional program that concurrently suppresses lipogenesis and promotes lipolysis. Collectively, these results provide mechanistic insight into how taurine alleviates obesity and support its further development as a novel therapy targeting the eCB-CB1 axis.

## Data Availability

The datasets for this study can be found in the Figshare repository (https://doi.org/10.6084/m9.figshare.31007782).

## Glossary

2-AG: 2-arachidonoyl glycerol
3D: three-dimensional
AA: arachidonic acid
AEA: anandamide
AMPK: 5’-AMP-activated protein kinase
CB1: cannabinoid receptor type-1
DAGL-α/β: diacylglycerol lipase-alpha/beta
DDA: data-dependent acquisition
DG: diacylglycerol
eCB: endocannabinoid
eWAT: epididymal white adipose tissue
FAAH: fatty acid amide hydrolase
FAS: fatty acid synthase
FBS: Fetal Bovine Serum
GP: glycerophospholipid
GPEtn: glycerophosphoethanolamine
HDL-C: high-density lipoprotein cholesterol
HFD: high-fat diet
IBMX: 3-Isobutyl-1-methylxanthine
LDL-C: low-density lipoprotein cholesterol
M/Z: mass-to-charge ratio
NAPE-PLD: NAPE phospholipase D
NCS: newborn calf serum
OPLS-DA: orthogonal partial least squares discriminant analysis
PCA: principal component analysis
PE: phosphatidylethanolamine
PGC-1α: PPAR*γ* coactivator-1α
PPAR-α: peroxisome proliferator-activated receptor-alpha
PPAR-γ: peroxisome proliferator-activated receptor-gamma
QC: quality control
RSD: relative standard deviation
RT: retention time
SREBP-1c: sterol regulatory element-binding protein-1c
T3: thyroxine
Tau: taurine
TC: total cholesterol
TG: triglycerides
TIC: total ion chromatogram
UCP1: uncoupling protein 1
VIP: variable importance in projection
WAT: white adipose tissue
