# Supplementary figures and images for: Taurine attenuates lipid accumulation via the eCB-CB1 axis: evidence from adipose metabolomics in HFD-fed mice and 3D adipocyte spheroids
Source: Front Nutr. 2026 Mar 6;13:1782392. doi: 10.3389/fnut.2026.1782392 (PMC13002624; doi:10.3389/fnut.2026.1782392)

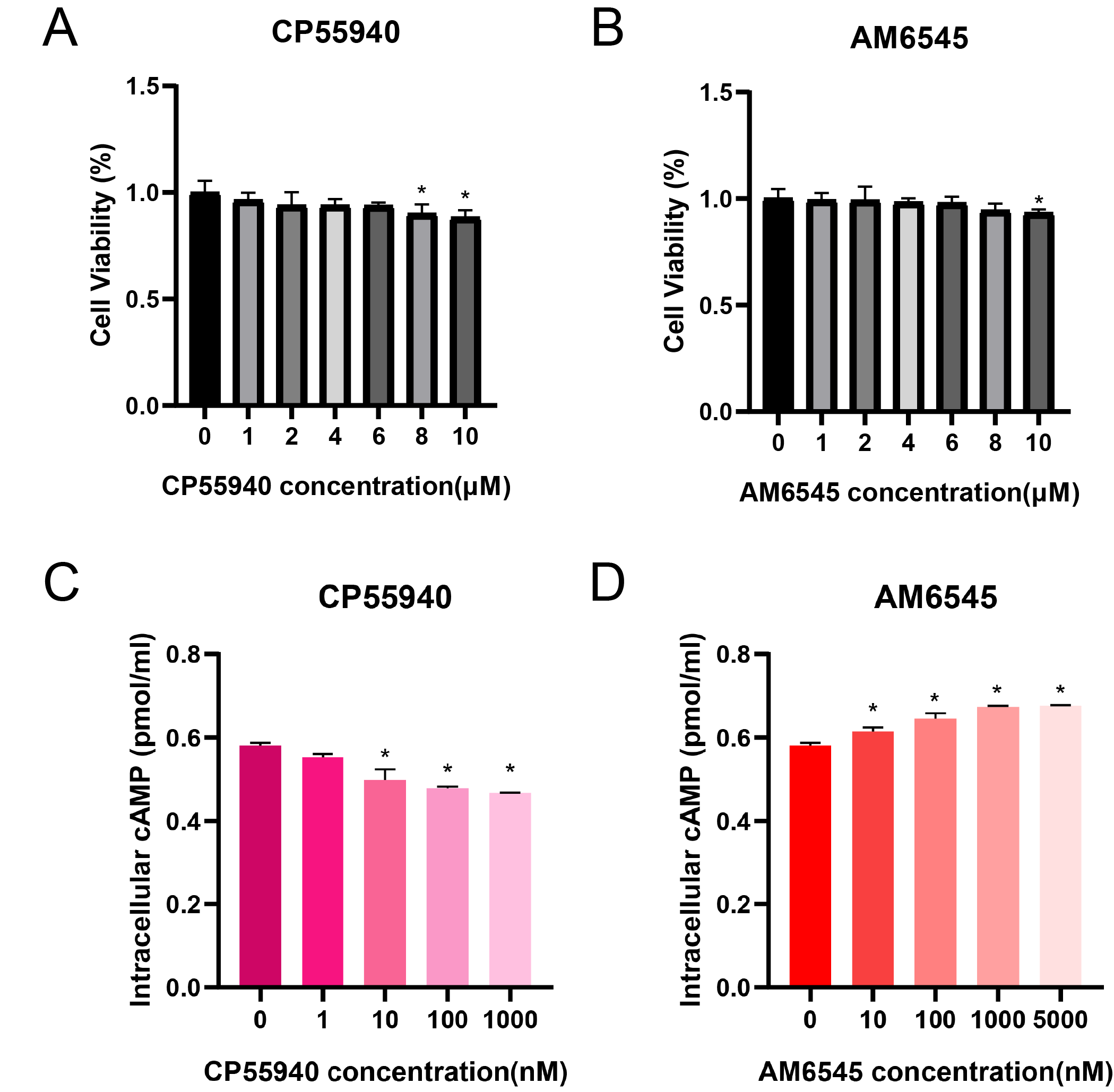

Supplement: Supplementary file 1 [file Image_1.png]

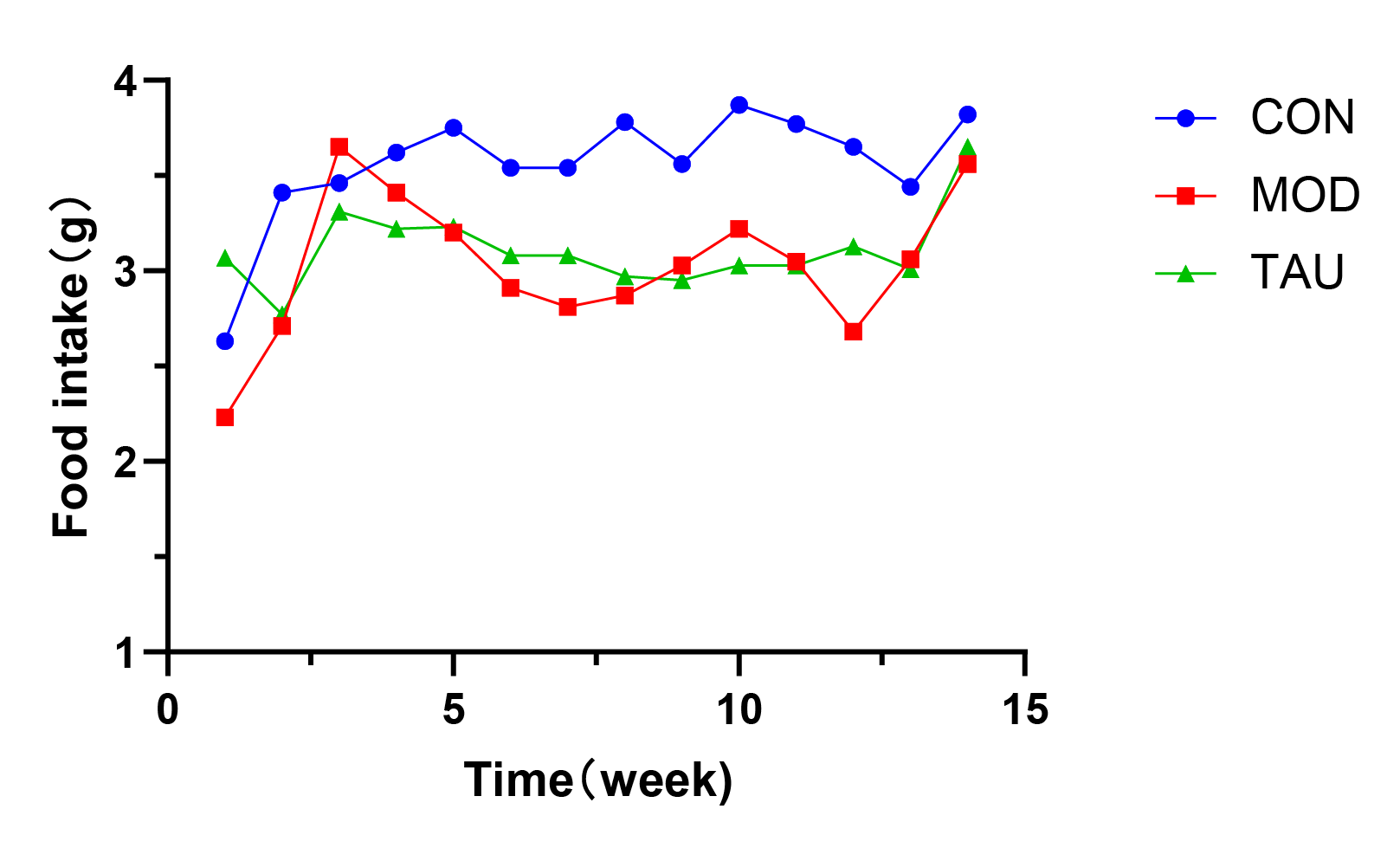

Supplement: Supplementary file 2 [file Image_2.png]

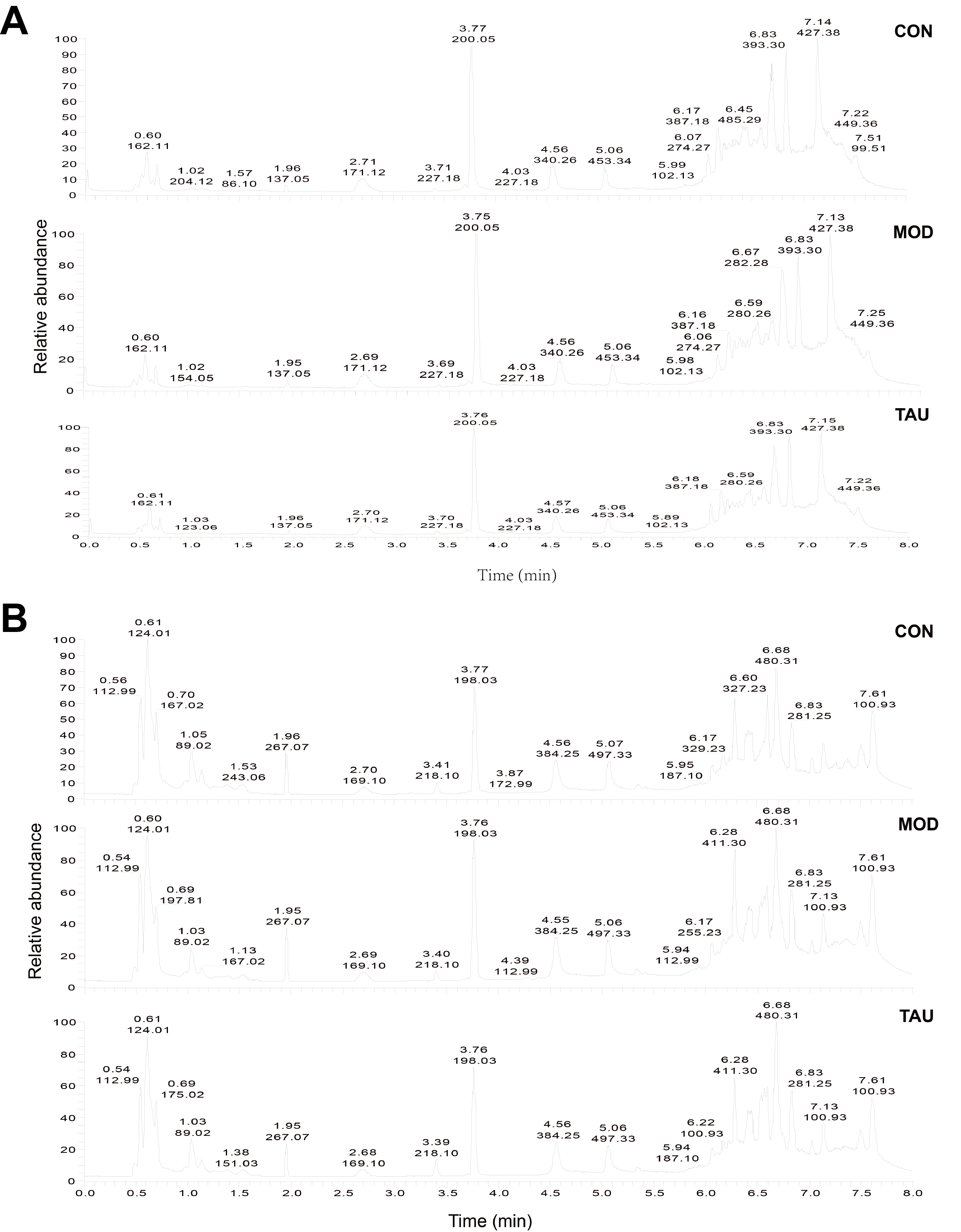

Supplement: Supplementary file 3 [file Image_3.png]

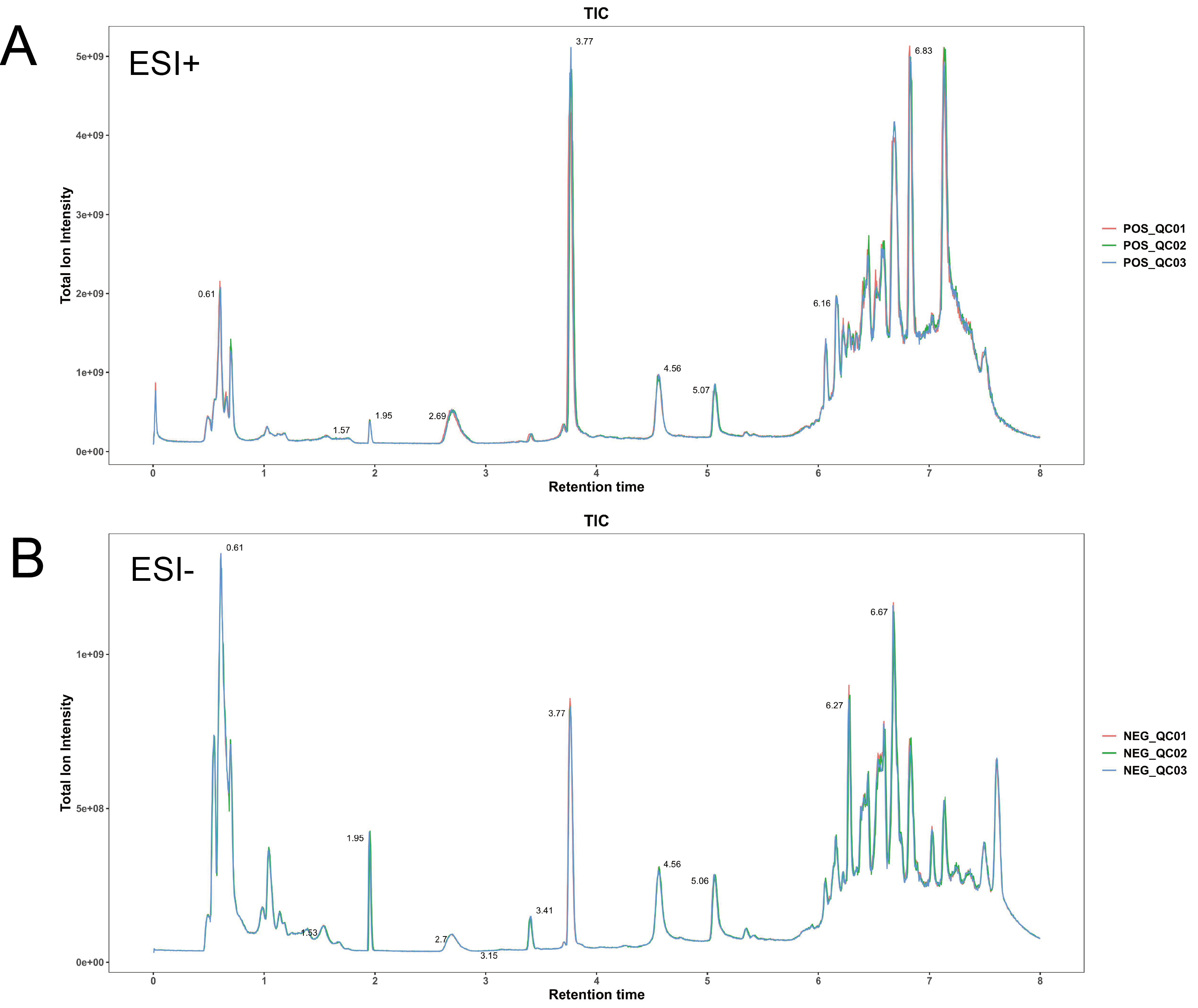

Supplement: Supplementary file 4 [file Image_4.png]

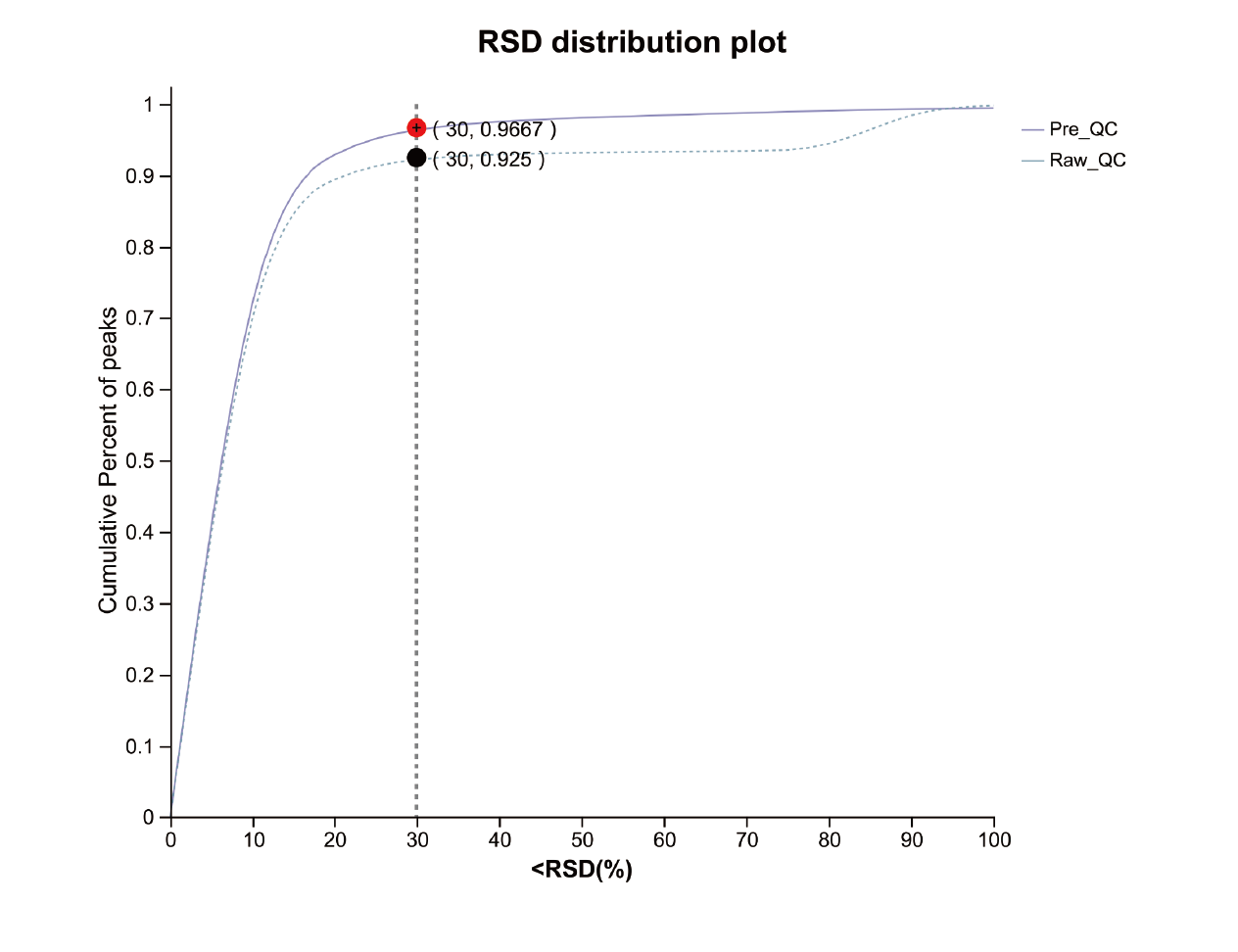

Supplement: Supplementary file 5 [file Image_5.png]

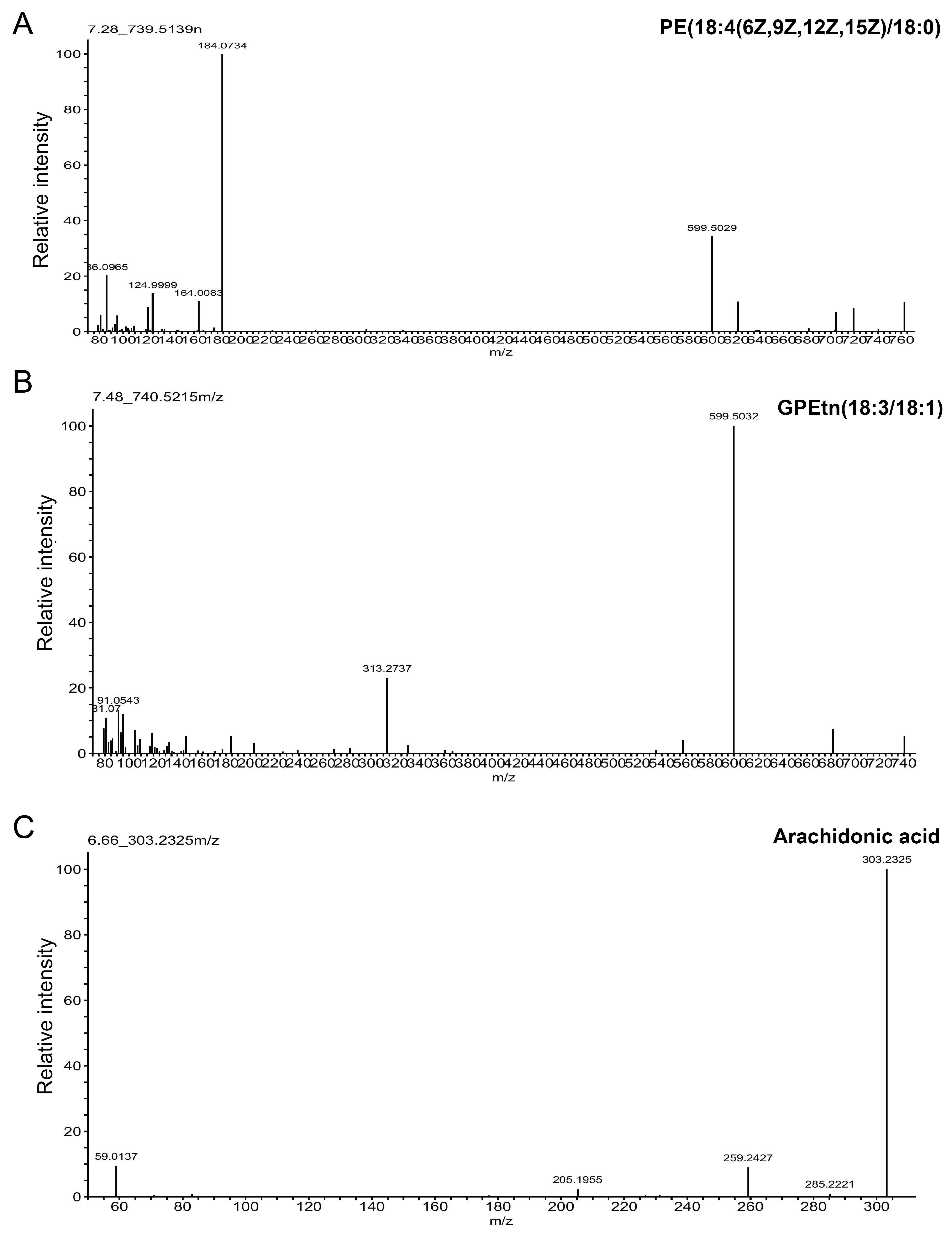

Supplement: Supplementary file 6 [file Image_6.png]
